# Chronic Traumatic Encephalopathy: A Comprehensive Narrative Review of Its Biomarkers

**DOI:** 10.7759/cureus.69510

**Published:** 2024-09-16

**Authors:** Aleena Majeed, Nageen Naz, FNU Namal, Sohaira Tahir, Vikash Kumar Karmani

**Affiliations:** 1 Internal Medicine, Fatima Jinnah Medical University, Lahore, PAK; 2 Internal Medicine, Social Security Hospital, Faisalabad, PAK; 3 Internal Medicine, Avicenna Medical College, Lahore, PAK; 4 Internal Medicine, Jinnah Sindh Medical University, Karachi, PAK

**Keywords:** chronic traumatic encephalopathy (cte), clinical biomarker, imaging biomarker, serum biomarker, traumatic brain injury

## Abstract

Chronic traumatic encephalopathy (CTE) is a progressive and fatal neurological disorder linked to repeated traumatic brain injuries (TBIs), including concussions and blows to the head. This condition is characterized by the accumulation of abnormally structured hyperphosphorylated tau proteins (p-tau), forming neurofibrillary tangles, astrocytic tangles, and neurites in the brain. CTE is often diagnosed post-mortem, making it challenging to diagnose and predict its progression in living individuals. Despite recent advancements, no definitive pathological, radiological, or neurobiological marker consistently shows promise in diagnosing and predicting the disease. This review aims to summarize the available techniques and advancements in imaging-based, genetic, neuropsychological, and fluid biomarkers for CTE, evaluating their specificity and sensitivity. It will also highlight the limitations of each marker in diagnosing CTE and provide future research directions to enhance the accuracy of CTE diagnosis in living individuals.

## Introduction and background

Sports-related traumatic brain injury (TBI) has gained prominence in the scientific literature over recent decades. Martland's 1928 description of boxers exhibiting behaviors like being "cuckoo" or "goofy" after head trauma introduced the term "punch drunk" for this syndrome. This recognition later led to "dementia pugilistica" for the consequences of repetitive brain trauma [1]. The now more commonly used term chronic traumatic encephalopathy (CTE) was coined in the 1940s. The exact number of people living with TBI-related long-term disability is unknown. Still, annual TBI cases are estimated at approximately 69 million worldwide, with the majority being mild (81%) and moderate (11%) in severity. The highest per capita incidence is in the United States and Canada (1299 per 100,000) and Europe (1012 per 100,000), while the greatest burden of head injury is in the Southeast Asian region (18.3 million) and the Western Pacific region (17.3 million) [2].

A blow or injury to the head can lead to TBI, which is classified as mild, moderate, or severe based on the patient’s Glasgow Coma Scale (GCS). A GCS score of 13-15 indicates mild TBI, 9-12 represents moderate TBI, and a score between 3 and 8 signifies severe TBI. CTE may develop from repeated head trauma, leading to the progressive degeneration of brain tissue, including the accumulation of an abnormal protein called tau. These brain changes can manifest months, years, or even decades after the last injury [3]. Early medical intervention in suspected cases of CTE aims to reduce suffering and slow disease progression, potentially extending healthy life years by delaying symptom onset and worsening. However, due to the absence of clinically approved biomarkers for CTE, diagnosing and intervening in the prodromal or early stages of the disease is currently impossible. Currently, CTE cannot be diagnosed in living individuals and can only be identified post-mortem through brain tissue analysis.

## Review

Genetic markers of CTE

Various factors such as duration of sports involvement and genetic predispositions impact the severity of tau pathology associated with the manifestation of CTE. However, the precise influence of these factors on gene expression and whether these effects persist consistently throughout the progression of the disease are yet to be elucidated.

Transmembrane Protein 106B (TMEM106B)

TMEM106B found in the endosomal and lysosomal membranes, facilitates ATPase binding activity and is involved in dendrite morphogenesis and lysosome localization. It is a key gene linked to CTE and is one of the first genetic factors identified. The gene burden can help explain why some athletes show severe CTE symptoms while others have milder effects despite similar head trauma levels. In athletes who have developed CTE, those carrying the "risk" variation of TMEM106B face a notably higher likelihood of experiencing dementia and elevated levels of p-tau [4].

APOE-ε4

Evidence now suggests that APOE-ε4 is linked to an increased risk of Aβ protein deposition in vivo [5]. Moreover, APOE is associated with hindered growth and branching of neurites in cell cultures. It is an effect mediated by the LDL receptor-related protein, which facilitates its entry into neurons and is upregulated post-injury. Compared to other APOE isoforms, APOE-ε4 exhibits weaker binding to cytoskeletal proteins and amyloid β protein, potentially diminishing any protective effects. Additionally, APOE-ε4 accelerates the aggregation of Aβ protein into amyloid fibrils in vitro.

Micro-RNAs (miRNAs)

MiRNAs are small RNA molecules that regulate various cellular processes at the post-transcriptional level. Changes in miRNA expression can occur in response to different physiological and pathological conditions. Studies have identified specific miRNA biomarkers, i.e., MiR-100-5p and MiR-10b-5p, that are increased in CTE [6]. Additionally, comparing the miRNA profiles in the biological fluids of individuals with a history of TBI and controls has identified differences in miRNA levels, indicating their potential as diagnostic biomarkers for CTE.

Strengths and Limitations

The importance of genetics in shaping the pathology and prognosis of CTE requires thorough examination. One advantage of miRNAs over traditional protein biomarkers is their stability; they show minimal changes even after exposure to room temperature for several hours or after freeze-thaw cycles. Quantifying target miRNAs using conventional polymerase chain reaction (PCR) methods is relatively straightforward, avoiding the need for highly sensitive assays or complex extraction processes required for protein quantification. With still limited research, it's unclear which miRNAs are most promising for diagnosing CTE. Moreover, one limitation of APOE-ε4 is its association with Alzheimer's disease (AD), which makes it a non-specific marker for CTE. Additionally, our understanding of the molecular mechanisms underlying CTE and its related brain pathology post-mortem remains incomplete.

Neuropsychological markers of CTE

Despite the extensive literature on the neuropathology of CTE, the comprehension of its neuropsychiatric markers remains enigmatic, possibly due to the non-specific nature of symptoms. The syndrome is thought to be related to one or more concussions, with symptoms ranging from mild to severe mental and physical decline, and in some cases, death. These encompass speech and gait difficulties, cognitive confusion, vertigo, and mental deterioration necessitating psychiatric care. Postmortem interviews with informants revealed impulsivity, depression, suicidality, anxiety, gait disturbances, and motor slowness in CTE subjects [7]. Multiple factors complicate the link between CTE and neuropsychiatric symptoms. At-risk individuals, often exposed to stressors like sports, combat, or illness, may undergo behavioral changes independently, making it challenging to differentiate pre-existing conditions from those developing after the onset of neurodegenerative disease.

Anxiety Disorders

Emotional processing and mood regulation involve complex interactions between prefrontal regions (e.g., anterior cingulate gyrus, orbitofrontal cortex) and limbic structures (e.g., amygdala, hippocampus, and ventral striatum) [8]. Traumatic lesions, such as diffuse axonal injury (DAI) and cerebral contusions can disrupt these neural circuits leading to affective disturbances that may persist and evolve. CTE is associated with anxiety, supported by psychological and neuropsychological evidence. Chronic anxiety is notably linked with right hemispheric cortical lesions resulting from brain trauma [9]. Conversely, anxiety can also impact the prognosis and recovery course of individuals with mild TBI.

Affective Disorder With Irritability

Major depression stands out as one of the most prevalent neuropsychiatric indicators observed in individuals post-CTE. Studies indicated that brain imaging shortly after head trauma revealed left dorsolateral frontal lesions and/or left basal ganglia lesions, which were identified as significant risk factors for major depression in CTE [10]. Persistent feelings of sadness, dysphoria, sleep disturbances, and feelings of guilt or low self-esteem collectively characterize depression as a biomarker of CTE. In a comprehensive case review study, dysphoria was noted in 48% of the cases, and apathy was observed in 9% of them among 51 neuropathologically confirmed cases of CTE, reviewed by McKee and colleagues [11]. In a 2017 review of 26 studies on TBI, researchers identified predictors for major depressive disorder (MDD), including female gender and pre-existing depressive symptoms [12].

Suicidality and Impulsivity

Suicide is a significant aspect of CTE, often reported in case series, reports, and reviews. Neuropathological features, such as cavum septum pellucidum (CSP), enlarged ventricles, and amyloid deposits, are linked to depression and increased suicide risk among former athletes and military veterans [13]. Omalu et al. reported that out of 17 subjects diagnosed with CTE postmortem, five died by suicide, while nine deaths were accidental, with five involving drug overdoses [14]. Behavioral changes, including heightened aggression and impulsivity, are common in CTE, with impulsivity seen in 82% of cases in the Mez et al. series. Damage to the hippocampal-septohypothalamic-mesencephalic circuitry or pathological changes in the amygdala and medial temporal lobe (MTL) may contribute to these behaviors. Frontal lobe atrophy and orbitofrontal pathology are associated with poor impulse control and disinhibition in individuals with CTE [15].

Post-traumatic Stress Disorder (PTSD)

A lot of research has focused on the link between TBI and PTSD. One study found that 27% of the sub-sample not unconscious for an extended period had current PTSD, while only 3% of those unconscious for more than 12 hours were diagnosed with PTSD. This suggests that PTSD may occur in TBI patients, particularly those who remained conscious during the accident, indicating a protective role of loss of consciousness against PTSD development [16].

Obsessive-Compulsive Disorder (OCD)

Behavioral changes and impairments of executive function were very common following TBI. OCD is characterized by recurrent and persistent thoughts, images, or impulses (obsessions) and repetitive behaviors or mental acts (compulsions) in response to the obsessions. In a study, OCD severity ranged from moderate to severe, with all patients experiencing multiple obsessions and compulsions. There was a high frequency of aggression, contamination, symmetry/exactness, somatic, and sexual obsessions, as well as cleaning, checking, and repeating compulsions. Unusual features such as obsessional slowness and compulsive exercising were also documented [17].

Strengths and Limitations

In essence, CTE is a complex condition marked by a range of neuropsychiatric symptoms linked to repeated head injuries. Despite progress in understanding its brain pathology, decoding its neuropsychiatric indicators remains challenging, spanning from depression and anxiety to impulsivity and aggression. The high suicide rate underscores its seriousness, urging continued research for better understanding and treatment. Given CTE's diverse neuropsychiatric profile, a holistic approach is crucial for diagnosis, management, and prevention.

Fluid biomarkers of CTE

A fluid biomarker refers to a molecule, biological activity, or concentration present in biological fluids, reflecting physiological or pathological processes occurring within the organism. Biomarkers for CTE can be detected in biofluids such as peripheral blood or cerebrospinal fluid (CSF). However, protein changes indicative of brain pathology have not been observed in readily accessible fluids like saliva, urine, or tears.

Markers of Inflammation

Following TBI, there is an observed increase in microglial activation, leading to the upregulation of various pro-inflammatory cytokines. These cytokines contribute to the disruption of the blood-brain barrier (BBB), enhanced secretion of chemokines attracting peripheral leukocytes to the brain, and the generation of reactive oxygen species (ROS), collectively promoting neuroinflammation and potentially triggering secondary cell death. Notably, the cytokines interleukin (IL)-6, IL-10, and tumor necrotic factor alpha (TNF-α), primarily secreted by microglia, have been investigated in the context of long-term TBI consequences, indicating microgliosis [18]. Goetzl et al. reported increased IL-6 levels in high-impact sports students and military veterans with TBI and cognitive impairment symptoms [19].

Markers of Neuronal Damage

Tau*: *Tau is a structural protein crucial for supporting microtubules (MT) in all body tissues. Within neurons, its primary function is to facilitate the proper assembly and structural stability of axonal MT. Following mTBI, an imbalance arises between kinases and phosphatases, leading to the accumulation of p-tau in the intracellular cytoplasm. This phosphorylation disrupts tau's ability to effectively bind to MT, resulting in the formation of neurofibrillary tangles (NFT) and neuropil threads in the cortex, which are key indicators for diagnosing CTE. Perivascular foci of P-tau in the neocortex and at the depths of the sulci are the most predictive of CTE. The amount of total tau (t-tau) in blood correlates with a compromised BBB, while p-tau aligns with signaling pathways affected by a compromised BBB, indicating the presence of DAI and neurotoxic mechanisms [20].

In a recent study, 96 ex-National Football League (NFL) players (aged 40-69) and 25 controls were examined showing that the plasma T-tau levels correlated positively with repetitive head impacts [21]. Another study, involving 78 former NFL players and 16 controls from the same cohort, conducted by Stern et al., revealed initial findings of tau-positive exosomes in plasma. These higher levels of exosomal tau were linked to poor performance on memory and psychomotor speed tests among the NFL group [22].

Neurofilament light (NF-L): NF-L combines with other neurofilament chains to construct the axonal cytoskeleton, particularly in large, myelinated axons extending into the subcortical white matter (WM) regions. In instances of trauma, these neurofilaments are released into the CSF and bloodstream.

Studies on football players show that NF-L serum levels rise during the season, especially one hour and one month after matches compared to controls, indicating possible axonal damage from repeated head impacts. These increased levels of plasma NF-L were associated with cognitive impairment and the severity of phosphorylated tau NFT (p-tau NFT) pathology post-mortem [23]. This suggests that NF-L could serve as a useful prognostic indicator for progressive neurodegeneration.

Amyloid β: Amyloid β plaques, primarily associated with AD, have been found to increase in concentration in brain tissue and CSF following TBI. These proteins contribute to the formation of plaques, which are detrimental to brain cells and can instigate neurodegenerative processes. The presence of Aβ plaques has been observed in individuals diagnosed with CTE and linked to factors such as possession of the ApoE4 allele and older age at death, suggesting a potential association between Aβ plaques and aging in CTE.

Studies have reported significant increases in Aβ40 and Aβ42 in military personnel with a history of TBI. Exosomal Aβ peptides show more promise, with significant increases observed in groups with TBIs [24].

Neuron-specific enolase (NSE):* *NSE, a glycolytic enzyme, shows increased levels in the bloodstream after neuron damage or demise. In sports involving head injuries, a study involving boxers revealed that serum levels of NSE remained elevated for two months following a match [25]. However, in ice hockey players who experienced concussions, serum NSE levels did not show an increase compared to preseason levels during the acute recovery phase [26]. In summary, while NSE has shown efficacy in detecting moderate and severe TBI, blood markers for mTBI do not appear to be sufficiently sensitive for practical application.

Ubiquitin carboxyl-terminal hydrolase L1 (UCH-L1): UCH-L1 is a crucial protein in the brain, playing a vital role in maintaining axonal integrity. Its malfunction has been linked to neurodegeneration, where it can contribute to the formation of NFT. Elevated levels of UCH-L1 have been observed in the CSF of Alzheimer's patients. In the context of potential CTE, CSF levels of UCH-L1 were associated with abnormalities in gray matter among long-term survivors of TBI. However, there have been no significant differences in UCH-L1 levels between TBI patients and controls, whether measured in plasma or exosomes [27], in existing studies.

Marker of Astrocyte Damage

Glial fibrillary acidic protein (GFAP): GFAP, an intermediate filament protein, is a major cytoskeletal component within astrocytes, contributing to the maintenance of synaptic transmission and axonal metabolism. Increased expression of GFAP accompanies astrocyte immune activation, while astrocytic injury can lead to the release of GFAP. Consequently, GFAP levels may reflect both chronic neuroinflammation and neurodegeneration in CTE. Shahim et al. found that serum GFAP significantly increased in chronic TBI patients up to five years after a single TBI compared to controls, though it showed limited association with structural brain changes [28].

Strengths and Limitations

Fluid biomarkers play a crucial role in various aspects of brain trauma management, including detecting injuries in asymptomatic individuals, differentiating between injury types, and monitoring recovery, disease progression, and treatment response. CSF offers unique advantages as a biomarker source due to its direct communication with brain interstitial fluid, low protease activity, and representation of brain biochemistry. Blood, on the other hand, interacts with brain and CSF compartments through the glymphatic system, providing easier access for serial biomarker sampling and potentially enabling rapid diagnosis, continuous monitoring, and improved treatment outcomes.

Despite the potential benefits, utilizing CSF for biomarker analysis involves the invasive procedure of lumbar puncture (LP), requiring specialized skills from physicians. In contrast, while blood is more accessible, measuring CNS-derived biomarkers in blood poses several challenges including the selective nature of the BBB, potential interference from other blood proteins, and issues with heterophilic antibodies and rapid protein degradation, thus limiting the accuracy of biomarker assessment in blood samples.

Neuroimaging markers of CTE

Neuroimaging plays a promising and guiding role in identifying biomarkers for CTE, among other indicators. Several neuroimaging biomarkers, including volumetric magnetic resonance imaging (MRI), diffusion tensor imaging (DTI), positron emission tomography (PET) scans, and electrophysiology, have been identified for CTE.

Computerized Tomography (CT) Scan

CT scans efficiently visualize fractures, bleeding (hemorrhage), blood clots (hematoma), brain tissue bruising (contusions), and swelling. However, they lack the resolution to detect microscopic changes specific to acute or chronic CTE in the brain.

*MRI*

Identifying structural MRI signatures of CTE is clinically crucial. Preliminary studies focusing on high-risk populations, such as former football players and fighters have shown consistent patterns, including reduced volumes in the frontal and temporal lobes, diminished MTL volume, increased shearing of WM fibers, and elevated prevalence, grade, and strength of CSP [29].

DTI

Biomechanical models suggest that rotational forces resulting from closed-head injuries can harm the WM fibers, causing axonal twisting and shearing. This may trigger secondary degenerative processes that develop over time. Over the past decade, DTI has become a crucial tool for evaluating WM bundle integrity in patients with TBI, including concussed athletes. It operates by analyzing the diffusion properties of water molecules, which provide insights into the micro-structure and organization of neural fibers. This method enables the assessment of structural integrity within precise regions of the brain in real time. By capturing these subtle alterations in vivo, DTI enhances our understanding of various neurological conditions and facilitates the development of targeted interventions for patients.

DTI relies on two fundamental measures that serve as crucial indicators of WM integrity: fractional anisotropy (FA) and mean diffusivity (MD).

FA measures the directionality of water diffusion in brain tissue, revealing the organization and coherence of neural fibers. Higher FA values typically indicate well-organized and intact WM tracts, whereas lower FA values suggest disruptions or abnormalities in fiber structure. Alternatively, MD indicates tissue density and cellularity by measuring overall water diffusion within a tissue. Elevated MD values often indicate increased water diffusion, which can result from various pathological processes such as inflammation, edema, or axonal damage [30].

In summary, decreased FA and elevated MD serve as important biomarkers of compromised WM integrity in CTE.

PET Scan

PET provides a real-time understanding of both typical and aberrant brain function on a molecular scale. It measures metabolic activities through radiopharmaceuticals tagged with short-lived positron-emitters such as carbon-11 (C11) or fluorine-18 (F18). These emit positrons which decay, generating photons captured by PET scanners. In CTE, there's a rise in Amyloid β (Aβ) deposition within the cortical gray matter and striatum. Using PET to target these plaques can reveal the temporal evolution of Aβ deposition post-TBI and its clinical implications, offering crucial insights into CTE pathology and potential diagnostic and therapeutic approaches.

Electroencephalography (EEG)

EEG measures electrical activity in the brain via electrodes. When an individual performs a cognitive task, the EEG records the neural activity as a series of waveforms. This scalp-recorded neural activity is referred to as event-related potentials (ERPs). ERPs are sensitive to concussion effects, revealing cognitive issues post-recovery. They detect changes in attention and memory, worsened by repeated concussions, even in asymptomatic athletes. Resting quantitative electroencephalography (qEEG) is a quantitative analysis of the EEG record, where the data are digitally coded and subjected to statistical analysis using the Fourier transform algorithm [31]. Distinct patterns of changes in quantitative features of the brain's electrical activity have been demonstrated to be sensitive to brain changes seen in neurodegenerative disorders and TBI. While these qEEG biomarkers are highly sensitive at the time of injury, the long-term effects of exposure to repetitive head injury on brain electrical activity are relatively unexplored.

Strengths and Limitations

Continuous EEG advancements offer deeper insights into cognitive shifts, highlighting the cumulative impact of repeated injuries over time. However, conventional MRI and CT scans lack the resolution to detect microscopic damage characteristic of DAI, thus limiting their ability to identify subtle brain parenchymal changes post-injury. Additionally, routine neuroimaging modalities cannot assess crucial factors like cerebral perfusion, metabolite levels, and mechanical properties of the brain [32], essential for understanding CTE's pathological processes. Moreover, the absence of commercially available PET tracers capable of detecting the p-tau protein, a significant marker for CTE, restricts accurate diagnosis and monitoring of the condition's progression using PET scans.

Future directions

Future directions for CTE biomarkers include exploring genetic markers, refining neuropsychological assessments, identifying novel fluid biomarkers, and advancing imaging techniques. TMEM106B shows promise as a biomarker for severe outcomes in CTE, while miRNAs hold potential but require further investigation. Physiological biomarker research involves investigating exosomal concentrations of biomarkers like p-tau, NfL, GFAP, and inflammatory cytokines, correlating them with structural and functional abnormalities identified in imaging and neurobehavioral assessments. Additionally, developing a reliable radiotracer for tracking P-tau deposition in CTE is a focus, with challenges including penetration of cell membranes and selective binding. Interdisciplinary studies on neuropsychiatric markers should prioritize uncovering underlying mechanisms and risk factors, with collaboration among researchers, clinicians, and stakeholders essential for developing personalized interventions and preventive measures. Ultimately, this understanding will lead to targeted therapies and improved outcomes for individuals with CTE.

## Conclusions

Longitudinal and multidimensional cohort studies that combine various approaches are crucial to understanding the complex pathology of CTE and the diverse nature of TBI. These studies should aim to uncover the connections between biomarker levels, neuroimaging showing brain structure and function, and clinical outcomes over time. Achieving these objectives necessitates collaborative efforts, thorough data collection over extended periods, and rigorous validation of results, recognizing the multifaceted nature of CTE and its related neurological conditions.
